# CSF T-Tau/Aβ_42_ Predicts White Matter Microstructure in Healthy Adults at Risk for Alzheimer’s Disease

**DOI:** 10.1371/journal.pone.0037720

**Published:** 2012-06-06

**Authors:** Barbara B. Bendlin, Cynthia M. Carlsson, Sterling C. Johnson, Henrik Zetterberg, Kaj Blennow, Auriel A. Willette, Ozioma C. Okonkwo, Aparna Sodhi, Michele L. Ries, Alex C. Birdsill, Andrew L. Alexander, Howard A. Rowley, Luigi Puglielli, Sanjay Asthana, Mark A. Sager

**Affiliations:** 1 Geriatric Research, Education and Clinical Center (GRECC), William S. Middleton Memorial Veteran’s Hospital, Madison, Wisconsin, United States of America; 2 Wisconsin Alzheimer’s Disease Research Center, Department of Medicine, University of Wisconsin, Madison, Wisconsin, United States of America; 3 Institute of Neuroscience and Physiology, Department of Psychiatry and Neurochemistry, The Sahlgrenska Academy at University of Gothenburg, Sweden; 4 University of Wisconsin School of Medicine and Public Health, Department of Medical Physics, Madison, Wisconsin, United States of America; 5 University of Wisconsin School of Medicine and Public Health, Department of Psychiatry, Madison, Wisconsin, United States of America; 6 Waisman Laboratory for Brain Imaging and Behavior, Madison, Wisconsin, United States of America; 7 University of Wisconsin School of Medicine and Public Health, Department of Radiology, Madison, Wisconsin, United States of America; Beijing Normal University, Beijing, China

## Abstract

Cerebrospinal fluid (CSF) biomarkers T-Tau and Aβ_42_ are linked with Alzheimer’s disease (AD), yet little is known about the relationship between CSF biomarkers and structural brain alteration in healthy adults. In this study we examined the extent to which AD biomarkers measured in CSF predict brain microstructure indexed by diffusion tensor imaging (DTI) and volume indexed by T1-weighted imaging. Forty-three middle-aged adults with parental family history of AD received baseline lumbar puncture and MRI approximately 3.5 years later. Voxel-wise image analysis methods were used to test whether baseline CSF Aβ_42_, total tau (T-Tau), phosphorylated tau (P-Tau) and neurofilament light protein predicted brain microstructure as indexed by DTI and gray matter volume indexed by T1-weighted imaging. T-Tau and T-Tau/Aβ_42_ were widely correlated with indices of brain microstructure (mean, axial, and radial diffusivity), notably in white matter regions adjacent to gray matter structures affected in the earliest stages of AD. None of the CSF biomarkers were related to gray matter volume. Elevated P-Tau and P-Tau/Aβ_42_ levels were associated with lower recognition performance on the Rey Auditory Verbal Learning Test. Overall, the results suggest that CSF biomarkers are related to brain microstructure in healthy adults with elevated risk of developing AD. Furthermore, the results clearly suggest that early pathological changes in AD can be detected with DTI and occur not only in cortex, but also in white matter.

## Introduction

Distinguishing pathologically aging elders from those who will age normally is a key challenge in preventing Alzheimer’s disease (AD). Several cerebrospinal fluid (CSF) markers that are presumably related to the core pathology in AD show promise for early detection of the disease. Lower levels of CSF Aβ_42_, higher CSF total tau (T-Tau), and higher tau phosphorylated at threonine 181 (P-Tau_181_), as well as biomarker ratios (T-Tau/Aβ_42_ or P-Tau/Aβ_42_) distinguish patients from controls [1], [2], [3], [4], [5] and predict conversion from mild cognitive impairment (MCI) to AD [1], [2], [6], [7], [8], [9]. When evaluated in cognitively healthy individuals, these biomarkers also appear to be related to cognitive function [10], [11], and measures of brain health that include cortical thinning [12], ventricular expansion and atrophy [11], [13], [14], [15], [16].

While studies resulting from volumetric imaging have been very useful for revealing gross structural changes, it is possible that some of the earliest brain changes involved in AD are subtle and below the detection threshold for volumetric imaging. Sensitive to water molecule motion, maps derived from diffusion tensor imaging (DTI) [17] provide unique information on brain microstructure. Mean diffusivity (MD) provides an index of isotropic diffusion of water molecules, and fractional anisotropy (FA) provides a measure of the degree of diffusion anisotropy, both of which may be altered by tissue damage. Maps based on the principal diffusivities that compose the diffusion tensor may provide additional information on microstructural alterations. The principal diffusivity (λ1), or axial diffusivity, represents water diffusion that is parallel to axons and may be altered by axonal injury; radial diffusivity (an average of λ2 and λ3), represents water diffusion that is perpendicular to axonal fibers and is thus linked to the microstructure of myelin [18], [19], [20], [21], [22], [23], [24], [25], [26]. DTI has been used to measure disease-related changes in patients with AD [27], [28], [29], [30], [31], [32], [33], [34], [35], [36], [37], [38], [39], as well as alterations in people with MCI [37], [40], [41], [42], [43], [44], [45], [46]. Using DTI, microstructural differences have also been detected in presumed presymptomatic AD including participants with APOE4 genotype [47], [48], [49] family history of AD [50], [51], or a combination of both risk factors [52].

In the present study, we examined the relationship between brain microstructure indexed by DTI and proteins associated with brain health in CSF collected in healthy middle-aged and older adults from the Wisconsin Registry for Alzheimer’s Prevention [53]. Additionally, because T1-weighted imaging may inform upon atrophy, we assessed the extent to which biomarkers in CSF were related to volume as assessed via T1-weighted imaging. Based on their utility in distinguishing AD patients from controls [1], [2], [3], [4], [5], the CSF biomarkers considered in this study were T-Tau, P-Tau_181_, and Aβ_42_. In addition to these markers of AD pathology, we analyzed CSF for a structural protein of neurons that is predominantly localized in large-caliber axons (and thus likely sensitive to axonal degeneration in white matter): neurofilament light chain protein (NFL) [54]. We hypothesized that baseline CSF measures of these markers would be related to brain health as measured with DTI, especially in brain regions that are affected early in the AD process–principally temporal and cingulate gray and white matter. We expected higher T-Tau and P-Tau and lower Aβ_42_ in CSF would be related to lower FA, higher MD, and altered radial and axial diffusivity in AD-sensitive brain regions. Additionally, we expected that NFL, being sensitive to axonal alteration, would be especially related to axial diffusivity–reflecting altered axonal health. Although the brain regions affected in AD are well known, the relationship between CSF biomarkers and brain health is a relatively new area of study, accordingly we used voxel-wise analyses (corrected for multiple comparisons) [55] to assess relationships across the entire brain.

## Materials and Methods

Study procedures were approved by the University of Wisconsin Health Sciences Institutional Review Board and were in accordance with U.S. federal regulations. All participants provided written informed consent.

### Participants

We enrolled 47 participants, four of whom were excluded due to unexpected abnormalities found on their MRI scan by the reviewing radiologist (HAR). The remaining 43 middle- to older- aged participants were 37 years of age to 66 years of age at time of lumbar puncture and CSF collection (mean = 53.67, SD = 7.77); and 42 to 71 years of age at time of follow-up scan (mean = 57.61, SD = 8.03). There were 12 men and 31 women. As a group, participants were well educated (mean = 16.26 years, SD = 2.60). All participants were from the Wisconsin Registry for Alzheimer’s Prevention (WRAP) [53] and had at least one parent with AD. WRAP is a registry of cognitively normal adults who are followed longitudinally and comprise individuals who have at least one parent with late onset AD and a group of controls with no family history of AD [53]. To verify the diagnosis of AD in parents of WRAP participants, parental medical records (and autopsy reports where available) were reviewed by a multidisciplinary diagnostic consensus panel. A diagnosis of AD in parents was made using standard clinical criteria [56], [57]. There were no families included with known autosomal dominant mutations. In the final sample, three participants had parents who were diagnosed with AD at autopsy, while the remaining clinical diagnoses were confirmed during a consensus meeting. Twelve participants (28%) were carriers of the ε4 allele of the apolipoprotein E (APOE4) gene. Inclusion criteria for all subjects consisted of the following: prior visit for lumbar puncture, normal cognitive function determined by neuropsychological evaluation, no contraindications for MRI and a subsequent normal MRI scan, no current diagnosis of major psychiatric disease or other major medical conditions (e.g., diabetes, myocardial infarction, or recent history of cancer), and no history of head trauma.

### Neuropsychological Testing

As part of their participation in WRAP, participants received at least one comprehensive neuropsychological assessment. Neuropsychological testing occurred an average of 1.80 years (SD = 1.28 years) out from the MR scan and 2.06 years (SD = 1.20 years) out from their lumbar puncture. In order to screen for changes in cognitive status, participants who were >3 months out from a neuropsychological testing visit also received the Mini Mental State Examination (MMSE) [58] upon the day of scan. We prospectively identified a subset of tests to examine memory, executive function, and speed of processing. These tests were *BVMT (Brief Visuospatial Memory Test, Revised)* total raw score, and delayed recall, which index visuospatial learning and memory [59]; *COWAT* (Controlled Oral Word Association Test; [60]) seconds to complete/raw score, to assess verbal fluency; *Trail Making Test A & B*, to assess motor speed, sequencing, and vigilance and in Trails B, the additional functions of rapid set shifting, serial retention and integration, verbal problem solving, and planning [61]; Wechsler Adult Intelligence Scale Working Memory Index–a composite of arithmetic, digit span, and letter number sequencing [62]–to assess the ability to hold information in working memory; *RAVLT (Rey Auditory Verbal Learning test)* total over the five learning trials, recognition, and 20 minute delayed recall raw scores [63] to assess immediate and delayed verbal memory.

### CSF Collection

The CSF collection was performed via lumbar puncture at a baseline visit in a simvastatin trial [64] an average of 3.41 years prior to brain imaging (SD = 1.19 yrs). CSF samples were collected in the morning after a 12-hour overnight fast, aliquoted in sterile polypropylene collection tubes, and stored in a −80°C freezer. The samples were subsequently sent in one batch to the Sahlgrenska University Hospital at the University of Gothenburg in Sweden for analysis. CSF T-Tau, P-Tau_181_, and Aβ_42_ analysis was accomplished via xMAP technology and utilizing the INNO-BIA AkzBio3 kit (Innogenetics) as previously described [65]. The xMAP technology is based on flow cytometric separation of antibody-coated microspheres that are labeled with a specific mixture of two fluorescent dyes. xMAP allows for simultaneous measurement of several analytes in the same tube, has low intra- and inter-assay variability, and high reproducibility even at low concentrations. NFL was analyzed using the sandwich ELISA method described in [66] with a limit of quantification of 125 ng/L. All analyses were performed on one occasion by certified laboratory technicians. Intra-assay coefficients of variation were below 10% for all analytes.

### Magnetic Resonance Imaging

Participants were invited for brain imaging on the condition that baseline CSF samples were available for analysis. Participants were imaged on a General Electric 3.0 Tesla Discovery MR750 (Waukesha, WI) MRI system with an 8-channel head coil and parallel imaging (ASSET). DTI was acquired using a diffusion-weighted, spin-echo, single-shot, echo planar imaging pulse sequence in 40 encoding directions, B0 = 1300, with eight non-diffusion weighted reference images. The cerebrum was covered using contiguous 2.5 mm thick axial slices, FOV = 24 cm, TR = 8000, E = 67.8, matrix = 96×96, resulting in isotropic 2.5 mm voxels. High order shimming was performed prior to the DTI acquisition to optimize the homogeneity of the magnetic field across the brain and to minimize EPI distortions. A T1-weighted volume was acquired in the axial plane with a 3D fast spoiled gradient-echo (3D EFGRE) sequence using the following parameters: TI = 450 ms; TR = 8.1 ms; TE = 3.2 ms; flip angle = 12°; acquisition matrix = 256×256×156, FOV = 260 mm; slice thickness = 1.0 mm.

### MRI Processing

Diffusion-weighted DICOM images were converted into NIFTI format using AFNI (http://afni.nimh.nih.gov/). FA, MD, and lambda maps (λ1, λ2 & λ3) were generated via the FMRIB Software Library (FSL) (http://www.fmrib.ox.ac.uk/fsl/fdt/index.html using the following procedures: (1) image distortions in the DTI data caused by eddy currents were corrected; (2) estimation of diffusion tensors was achieved using DTIFIT; (3) three-dimensional maps of FA, MD, and the 3 eigenvalues (λ1, λ2 & λ3) were computed from the tensors from step (2). Each participant’s FA map was aligned to an FA template in Montreal Neurological Institute (MNI) space, comprised of an average of 121 FA maps acquired from healthy participants with similar demographics as the study cohort. Transformations were achieved via 12-parameter affine transformation and nonlinear deformation using Statistical Parametric Mapping software (SPM8 available at http://www.fil.ion.ucl.ac.uk/spm). Estimated transforms from each participant’s FA map warping were applied to the participant’s remaining DTI maps (MD, λ1, λ2 & λ3) resulting in a transformation of all the original images into MNI space. The normalized FA maps were used to visually inspect for accurate normalization using the “check registration” function in SPM and selecting specific fiber tracts for comparison (corpus callosum, cingulum, superior longitudinal fasciculus). λ2 & λ3 maps were averaged to create radial diffusivity maps, and λ1 was the axial diffusivity map.

Processing of the T1-weighted images was performed using a six class segmentation tool in SPM8. Processing involved bias correction and iterative normalization and segmentation of the original anatomic images [67] into distinct tissue classes (gray matter, white matter, cerebrospinal fluid, skull, fat tissue, and image background) using spatial prior information. Gray matter tissue segments were normalized to MNI template space via a 12-parameter affine transformation and nonlinear deformation (with a warp frequency cutoff of 25). The segmented and normalized gray matter maps were “modulated”, which involves scaling the final gray matter maps by the amount of contraction or expansion required to warp the images to the template. The final result was a gray matter probability map for each participant in which the total amount of gray matter remained the same as in the original images. The spatially normalized gray matter maps were smoothed using an 8-mm Gaussian kernel before being entered into the statistical analysis.

### Statistical Analysis

In order to test the relationship between CSF proteins, age, and cognitive function, we performed linear correlation analysis using SPSS (Release 19.0.0, Chicago, SPSS Inc.). CSF biomarkers were log transformed to normalize distribution prior to inclusion in analysis. Differences in CSF protein levels based on APOE status and gender were tested using independent t-tests in SPSS. In order to test the extent to which CSF biomarkers were associated with brain health as indexed by the DTI maps and T1-weighted imaging, voxel-wise linear regression models were implemented via statistical modules available in SPM8. The independent variables were Aβ_42_, total tau (T-Tau), phosphorylated tau (P-Tau_181_), T-Tau/Aβ_42_, and P-Tau_181_/Aβ_42_, while the dependent variables consisted of participants’ DTI maps and gray matter probability maps. Due to relatively low levels of NFL in the majority of participants (NFL<125 ng/L; N = 33), NFL was treated as a categorical variable. The detection limit of the assay is NFL<125 ng/L, thus we used this as a cut-point to determine the groups. Differences in brain health based on NFL was tested using voxel-wise ANCOVA, where DTI maps or gray matter probability maps were the dependent variable and the groups were NFL>125 ng/L, and NFL<125 ng/L. Age (at time of MR scan) and gender were included as covariates in all models due to known effects on brain microstructure [68], [69], [70], [71]. Because the imaging was performed in a group of participants that had been randomized in a simvastatin trial, only baseline (pre-simvastatin) CSF measures were used and a treatment vs. placebo covariate was included in the statistical models. All analyses were thresholded at p<.05 corrected for multiple comparisons using false discovery rate (FDR) correction [72]. In order to exclude small clusters and increase the anatomical plausibility of the results, a cluster size threshold of 50 contiguous voxels was used and analyses were restricted to gray and white matter using a binary brain mask. The binary brain mask was computed by thresholding the SPM brain mask in MNI space (which contains voxels that vary from 0 percent to 100 percent probability of brain) at a threshold of.5 or greater (50% probability or greater of being brain). Furthermore, analysis of the gray matter probability maps used an absolute threshold masking of 0.1 to exclude voxels with a low probability of being gray matter.

## Results

### Demographics, Neuropsychological Function, and CSF Biomarkers

There was a significant positive correlation (P<.05) between age at time of lumbar puncture and T-Tau, T-Tau/Aβ_42_, and P-Tau_181_/Aβ_42_ (Pearson correlation coefficients for age and CSF markers are shown in **Table 1**). Men and women did not differ on CSF biomarker levels of T-Tau, P-Tau_181_, Aβ_42_, T-Tau/Aβ_42_, or P-Tau_181_/Aβ_42_. There was a significant difference in T-Tau and Aβ_42_ between APOE4 positive and APOE4 negative participants, where T-Tau was higher in the non-carriers (m = 4.25) compared to carriers (m = 3.90), t(41) = 2.038, p<.05, and Aβ_42_ was lower in the carriers (m = 5.57) compared to non-carriers (m = 5.83), t(41) = 3.062, p<.05. APOE4 carriers and non-carriers did not differ on age (p = .78).

**Table 1:** Linear correlation among CSF biomarkers and age.

		Age	T-Tau	P-Tau_181_	Aβ_42_	T-Tau/Aβ_42_
Age	Pearson Correlation					
T-Tau	Pearson Correlation	0.367 *				
P-Tau_181_	Pearson Correlation	0.253	**0.585** Ŧ			
Aβ_42_	Pearson Correlation	−0.147	0.256	0.228		
T-Tau/Aβ_42_	Pearson Correlation	0.443 *	**0.856** Ŧ	0.459 *	−0.281	
P-Tau_181_/Aβ_42_	Pearson Correlation	0.326 *	0.436 *	**0.854** Ŧ	−0.310 *	**0.599** Ŧ

Significant correlation at P<.05 *.

Significant at p<.001 Ŧ.

All participants were cognitively normal as determined by comprehensive neuropsychological testing and as assessed by MMSE (MMSE ≥27). Controlling for age and education, linear correlation analysis indicated that baseline CSF biomarker levels were related to a subset of the neuropsychological test scores. WAIS Working Memory Index (scaled for age and education) was positively correlated with T-Tau (r = .307, p<.05) and with T-Tau/Aβ_42_ (r = 0.303, p = .051). However, a scatter-plot revealed that the participant with the highest tau value (>2 SD higher than the mean) performed well on working memory–skewing the results. When this participant was removed from the analysis, the relationships were no longer significant. RAVLT recognition score was negatively correlated with both P-Tau_181_ (r = −.379, p<.05) and P-Tau_181_/Aβ_42_ (r = −.362, p<.05) but was not related to T-Tau, Aβ_42_, or their ratio. No other RAVLT sub-scores or other neuropsychological tests were significantly correlated with the CSF markers.

### Imaging Results

**Figure 1:**
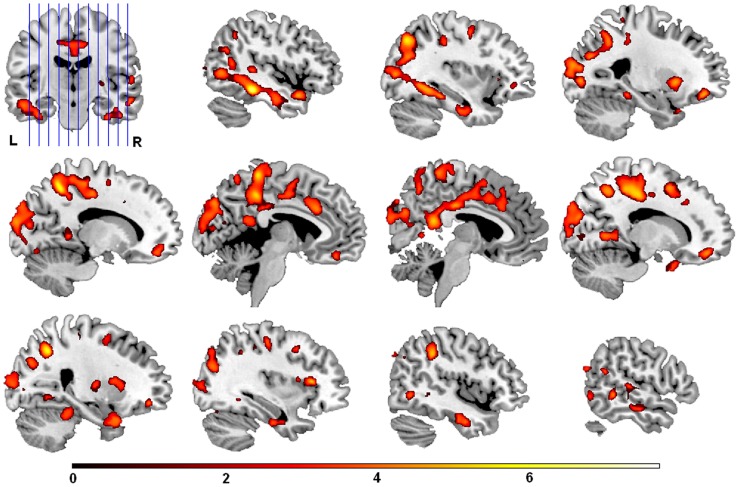
CSF T-Tau/Aβ42 and mean diffusivity. Higher T-Tau/Aβ42 at baseline was associated with increased mean diffusivity in follow-up scanning in several brain regions, encompassing both gray and white matter. As shown above, this relationship was especially prominent in temporal lobe white matter adjacent to hippocampus, but also encompassing gray and white matter in frontal and parietal lobes, portions of occipital white matter, and small clusters in cerebellum. Results are FDR corrected for multiple comparisons (p<.05) and displayed here with a cluster size threshold of 20 or more voxels. Sections are shown in sagittal view beginning from the left side of the brain to right. Variations in the color map reflect the size of the T-statistic (indexed by the color bar at bottom).

**Figure 2:**
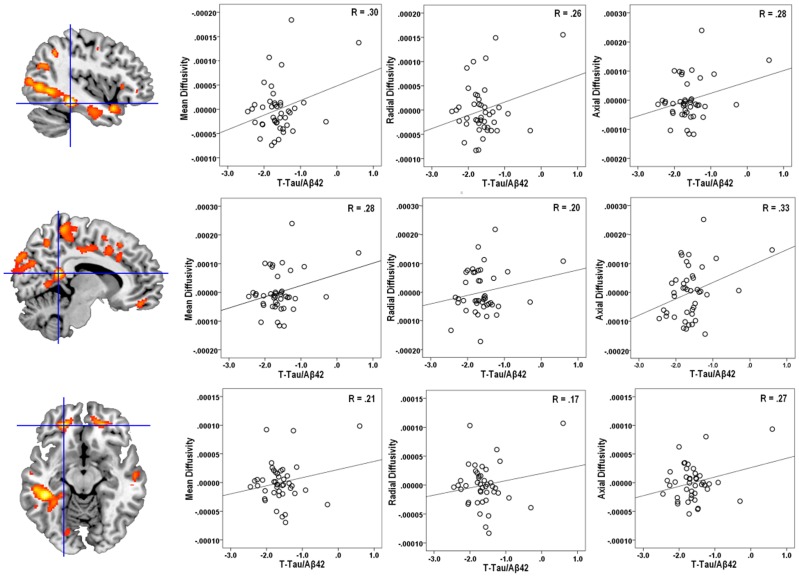
T-Tau/Aβ42 Plotted against mean, radial, and axial diffusivity. Shown here are the results of the voxel-wise analysis, where regions with color overlay are those where higher T-Tau/Aβ42 was associated with higher diffusivity (mean, radial, and axial). In order to illustrate the relationship between T-Tau/Aβ42 and the diffusivity maps, we extracted diffusion values from representative regions of significant correlation in the voxel-wise analysis and plotted them against T-Tau/Aβ42. Shown on the top row are diffusion values extracted from the left temporal lobe (x = −42, y = −34, z = −16) plotted against T-Tau/Aβ42. In the middle row are diffusion values extracted from right posterior cingulum bundle (x = 8, y = −46, z = 16) plotted against T-Tau/Aβ42. In the bottom row are diffusion values extracted from left inferior frontal white matter (x = −22, y = 43, z = −12) plotted against T-Tau/Aβ42. Blue crosshairs overlaid on the brain sections indicate the location of the extracted values. Each point in the scatter represents diffusion values from one participant (n = 43). T-Tau/Aβ42 values were log-transformed and mean, radial, and axial diffusivity values were adjusted for age at time of scan, sex, and treatment (CSF data were collected at baseline in a Simvastatin treatment trial, data from the prevention trial are not shown here).

Voxel-wise regression analysis indicated that both T-Tau and T-Tau/Aβ_42_ showed robust and widespread positive relationships with several of the DTI measures, specifically MD, axial and radial diffusion. These relationships were extensive in white matter and were prevalent in temporal, parietal and frontal lobes. The locations of peak T-value for MD and FA clusters obtained in the voxel-wise analysis are tabulated in **Table 2** (axial and radial diffusivity results are tabulated in **Table S1**). The brain regions where CSF T-Tau/Aβ_42_ predicted MD values in the voxel-wise regression analysis are shown in **Figure 1**; the positive relationship between T-Tau/Aβ_42_ and MD is shown in **Figure 2** in scatter plots from a subset of the significant clusters found in the voxel-wise regression analysis. The regional overlap between T-Tau and T-Tau/Aβ_42_ SPM result maps (MD, axial and radial diffusivity) was extensive, and is summarized in terms of percent overlap in **Table 3**.

**Table 2:** Regions where CSF biomarkers were significantly correlated with FA and MD in the voxel-wise analyses.

	MNI coordinatesx y z	Peak T value	k (mm^3^)
**T-Tau & Fractional Anisotropy**	×	×	×
**T-Tau & Mean Diffusivity**			
R Parietal Lobe WM	26, −59, 42	7.89	13890
R Medial Frontal Gyrus	16, 7, 52	6.37	478
L Middle Frontal Gyrus	−24, 43, −14	5.83	259
R Middle Temporal Gyrus	58, −42, −1	5.33	159
L Frontal Lobe, WM	−18, −4, 59	5.25	154
R Corpus Callosum	12, −45, 12	5.23	414
R Medial Frontal WM	12, 46, −18	5.21	207
L Superior Temporal Gyrus WM	−60, −23, 7	4.73	112
R Lentiform Nucleus, Putamen	26, −9, 7	4.45	256
R Superior Temporal Gyrus WM	60, −24, −1	4.39	145
L Temporal Lobe WM	−52, −13, −21	4.37	143
L Inferior Frontal Gyrus	−40, 25, 3	4.25	56
R Fusiform WM	38, −71, −21	4.06	83
R Entorhinal WM	20, 11, −28	3.99	110
R Lingual Gyrus	28, −61, −4	3.96	74
R Middle Temporal Gyrus	46, −63, −6	3.91	52
L Inferior Parietal Lobule	−52, −41, 40	3.75	63
**P-Tau & Fractional Anisotropy**	×	×	×
**P-Tau & Mean Diffusivity**	×	×	×
**Aβ_42_ & Fractional Anisotropy**			
L,R Medial Frontal Gyrus	0 11 −24	6.27	66
**Aβ_42_ & Mean Diffusivity**	×	×	×
**T-Tau/Aβ_42_ & Fractional Anisotropy**	×	×	×
**T-Tau/Aβ_42_ & Mean Diffusivity**			
L Temporal Lobe WM	−44 −36 −13	7.71	11374
R Uncus	20 9 −27	6.11	286
L Middle Frontal Gyrus WM	−22 43 −12	5.69	257
R Insula WM	36 26 11	5.50	229
R Supramarginal Gyrus WM	44 −39 39	5.17	225
R Medial Frontal Gyrus WM	12 46 −16	5.09	204
R Middle Temporal Gyrus	58 −42 −1	5.07	126
L Superior Temporal Gyrus	−42 13 −22	5.05	157
L Putamen	−24 14 0	5.01	100
R Middle Temporal Gyrus	48 −63 −4	4.87	105
R Superior Temporal Gyrus WM	58 −24 5	4.68	154
L Superior Temporal Gyrus WM	−62 −21 7	4.54	109
R Temporal Lobe WM	42 −6 −31	4.48	209
L Superior Temporal Gyrus WM	−62 −43 14	4.41	99
R Superior Occipital Gyrus WM	34 −77 26	4.23	409
R Occipital Lobe WM	28 −59 −4	4.20	87
R Middle Temporal Gyrus WM	56 −14 −16	4.04	76
R Precentral Gyrus WM	34 −22 51	3.91	100
L Frontal Lobe WM	−30 −35 39	3.82	100
R Cerebellum, Anterior Lobe	24 −37 −21	3.78	118
R Putamen	28 −10 3	3.76	82
**P-Tau/Aβ_42_ & Fractional Anisotropy**	×	×	×
**P-Tau/Aβ_42_ & Mean Diffusivity**	×	×	×
**NFL & Fractional Anisotropy**			
L Middle Temporal Gyrus WM	−42 −65 11	6.66	40
**NFL & Mean Diffusivity**	–	–	–

MNI: Montreal Neurological Institute; k: cluster size; T-Tau: Total Tau; P-Tau: Phosphorylated Tau; WM: White Matter; L: Left; R: Right.

× No relationship with any regions at FDR corrected threshold p<.05.

− No group differences in any region at FDR corrected threshold p<.05.

**Table 3:** Percent of regional overlap between statistical parametric mapping result maps.

	T-Tau & MD	T-Tau & Rad.	T-Tau & Ax.	T-Tau/Aβ_42_ & Ax.	T-Tau/Aβ_42_ & Rad.
T-Tau/Aβ_42_ & MD*	66%	74%	60%	85%	95%
T-Tau/Aβ_42_ & Rad. Diff.	57%	70%	49%	68%	
T-Tau/Aβ_42_ & Ax. Diff.	54%	58%	58%		
T-Tau & Ax. Diff.	84%	83%			
T-Tau & Rad. Diff.	70%				

All results maps were the product of a linear correlation analysis, where the CSF measures (T-Tau and T-Tau/Aβ42) were used to predict the diffusion measures (MD, axial and radial diffusivity). T-Tau: Total Tau; MD: Mean Diffusivity; Rad: Radial; Ax: Axial; Diff: Diffusivity.

* Result map shown in Figure 1.

At a statistical threshold of p<.05 (FDR corrected for multiple comparisons) there was no relationship between T-Tau or T-Tau/Aβ_42_ and FA. Furthermore, neither P-Tau_181_ nor P-Tau/Aβ_42_ were related to any of the DTI measures (FA, MD, axial or radial diffusivity). Higher Aβ_42_ in CSF was related to higher FA in medial frontal gyrus, but not related to MD, axial or radial diffusivity. Participants with NFL>125 ng/L showed a single region of higher FA in middle temporal gyrus white matter compared to control, with no differences in MD, axial or radial diffusivity. Complete results (cluster extent, T-value, and locations) for all CSF measures and FA and MD maps are in **Table 2** (axial and radial diffusivity results are tabulated in **Table S1**). None of the CSF biomarkers predicted gray matter volume as indexed by the modulated gray matter probability maps (FDR p<.05).

## Discussion

### General

AD-related CSF biomarkers are linked with global and regional brain volumes in healthy elderly [12], [13], [14]. In this study, we assessed the relationship between brain tissue microstructure measured with DTI and AD-related CSF biomarkers. Additionally, we assessed the relationship between CSF biomarkers and gray matter volume. Our group has previously found that cognitively healthy people with parental family history of AD show microstructural brain differences compared to those without risk for AD [50]. In this study, we found that CSF biomarkers previously associated with AD are related to brain tissue microstructure in similar regions as those previously found to be associated with parental family history.

While gray matter volume was not related to any of the CSF biomarkers in this study, CSF biomarkers were related to white matter health as indexed by DTI; interestingly, the regions of significant association were mostly *adjacent* to critical gray matter structures that are known to be affected in AD. This was especially true for T-Tau and T-Tau/Aβ_42_, where higher CSF levels were related to higher axial, radial, and mean diffusivity in a large swath of temporal lobe white matter adjacent to hippocampus. Several studies now point toward early involvement of white matter in AD development, including human studies on AD risk [48], [49], [50], [52], [73], and studies on a triple transgenic mouse model of AD indicating white matter changes may precede other measurable pathology in AD [74], [75]. In contrast, studies conducted in AD and memory impaired patients have found that CSF biomarker levels are related to gray matter structures including hippocampus, entorhinal cortex [76], and posterior cingulate cortex [77], One possibility is that early AD pathology involves axonal or myelin degeneration and that cortical change is only measurable at later disease stages or in more elderly individuals.

### White Matter Degeneration in AD: Myelin and Axons

White matter degeneration in diagnosed AD is confirmed by several human post mortem studies [78], [79], [80], [81]. MRI enabled ante mortem studies substantiate post mortem findings, showing decreased regional white matter volumes in AD compared to controls [82], [83], [84], [85], [86], [87], [88], [89], [90], [91], [92] and differences in water diffusion properties in white matter [27], [28], [29], [30], [31], [32], [33], [34], [35], [36], [37], [93]. White matter alterations in confirmed AD are a combination of axonal and myelin alteration, yet it is unknown whether white matter changes are secondary to damage of the neuronal soma, involve primary axonal degeneration [94], [95], are linked with myelin degeneration [39], [46], [96], [97], [98], or represent some combination of these events.

In our study we measured the relationship between CSF biomarkers and DTI measures that are presumably related to axonal health (axial diffusivity) and myelin health (radial diffusivity). We found that both T-Tau and T-Tau/Aβ_42_ predicted axial and radial diffusivity, suggesting these CSF biomarkers are potentially related to both neuronal axon integrity and health of oligodendrocyte synthesized myelin. Furthermore, the analyses of axial, radial, and mean diffusivity produced statistical maps that were highly overlapping, suggesting that alterations in one cell component (e.g. neuronal axons) could very well be associated with related alterations in another cell component (myelin).

Although CSF measures of myelin were not available to us in this study, we assessed axonal degeneration via CSF measured neurofilament light protein. Neurofilament proteins are major constituents of the axonal cytoskeleton, consisting of three polypeptides; light (NFL), medium and heavy subunits. NFL proteins are most related to large-caliber axons [99] and when axons are damaged, NFL is released into CSF. NFL proteins measured in CSF are significantly higher in AD compared to controls [66], [100], [101], [102], [103], [104]. In our study,a group comparison based on CSF NFL levels yielded a single region of difference in middle temporal gyrus white matter, where participants with higher CSF NFL showed higher FA. Although we expected that the group with presumably greater axonal degeneration would show lower FA, it’s possible that early stage axonal degeneration in the presence of intact myelin could lead to higher anisotropy. Another possibility is a loss of crossing fibers in this region–which could lead to higher FA. Although we did not find a relationship between CSF NFL and the mean, radial, and axial diffusivity maps at a relatively conservative statistical threshold, analyses performed at uncorrected (for multiple comparisons) thresholds did show differences between NFL groups. Interestingly, the group with higher CSF NFL showed higher axial diffusivity in cingulum and white matter adjacent to hippocampus. Because only ten participants in our study had NFL levels greater than the detection limit of 125 ng/L, we were likely underpowered to find these subtle differences at corrected thresholds and further work will be needed to expand upon these preliminary findings.

### Classic Pathology: Abnormal Phosphorylation of Tau and Amyloid Deposition

In AD, abnormal phosphorylation of tau protein and deposition of amyloid are considered primary processes underlying neuronal degeneration. Hyperphosphorylation of tau and its subsequent release from the cell means markers of tau measured in CSF increase compared to controls [105]. In contrast, CSF markers of amyloid decrease as amyloid is deposited in the brain [3], [4], [106], [107]. In our study, we found widespread relationships between elevated CSF T-Tau and altered measures of brain tissue microstructure indexed by mean, radial, and axial diffusivity, with somewhat similar but also distinctive patterns occurring for T-Tau/Aβ_42_ (up to 70% overlap in the radial diffusivity maps). Although the localized source of tau protein measured in CSF is unknown, higher levels in our study were related to higher diffusion across temporal, parietal, and frontal lobes–particularly in white matter. We speculate CSF tau protein may have originated from axons in these brain regions, but we can not rule out that tau may have also originated from oligodendroglia [108]. Phosphorylation of tau is associated with the development of glial tangles in human patients with AD [109], [110], [111], as well as in animals expressing mutant tau [112], [113], [114]. Interestingly, CSF levels of tau phosphorylated at threonine 181 did not show a relationship with any of the DTI measures at the p<.05_FDR_ threshold. Although some studies suggest that CSF P-Tau_181_ does not correlate with neuropathological burden in AD [115], in our study it is possible that the relationship between P-Tau_181_ and tissue microstructure was too subtle to survive our statistical threshold. Indeed, when we performed exploratory analyses at uncorrected (for multiple comparisons) thresholds, higher P-Tau_181_ was associated with altered tissue microstructure. Furthermore, P-Tau_181_ and P-Tau_181_/Aβ_42_ were the only biomarkers related to memory function in this study. Nonetheless, considering alternative sites of tau phosphorylation will likely be important for understanding early AD and in AD biomarker development.

While T-Tau and T-Tau/Aβ_42_ showed widespread relationships with the DTI measures, CSF Aβ_42_ was associated with lower anisotropy of water (FA) only in medial frontal cortex and underlying white matter. Deposition of brain amyloid in AD likely involves frontal involvement at the earliest stages [116], which presumably could have resulted in altered tissue microstructure in this study. Studies using amyloid imaging in this cohort are expected to shed further light on regional amyloid deposition in the earliest stages of AD.

### CSF Biomarkers in Cognitively Healthy Adults

In healthy older adults, levels of AD biomarkers measured in CSF are related to age, cognition, structural brain volume, and AD risk factors including APOE4 status [10], [117], [118] and family history [51]. In our sample of healthy adults with elevated risk for AD, we found significant positive correlations between age and T-Tau, T-Tau/Aβ_42_, and P-Tau_181_/Aβ_42_. Alone, Aβ_42_ was not related to age. We also found that both T-Tau and Aβ_42_ differed between APOE4 carriers and non-carriers. Similar to previous studies, Aβ_42_ was lower in the carriers compared to non-carriers–conceivably suggesting greater amyloid deposition in the group at greater risk for AD. Interesting, we found that the APOE4 carriers in this sample showed lower T-Tau compared to non-carriers. These T-Tau findings are consistent with our previous report on CSF biomarkers in middle-aged at-risk adults [119]. Although APOE4 has been linked with higher T-Tau in some studies [120], [121], our sample differs in that all of the participants were positive for parental family history of AD–a group which in middle age shows higher T-Tau levels than the general population [51]. Further studies will be needed to dissociate the effects of APOE and family history of AD on CSF biomarkers in middle-age.

All of the participants in our study tested cognitively normal. Among the cognitive tests, only recognition memory was related to any of the CSF measures. Participants with higher P-Tau_181_ and P-Tau_181_/Aβ_42_ levels had lower recognition on a list learning test (RAVLT). Glodzik et al. have also found a relationship between P-Tau (P-Tau_231_) and memory function [11], and combined use of P-Tau_181_ and Aβ_42_ has also been shown to predict longitudinal subjective memory impairment in healthy elderly [122]. Because our study was cross-sectional and restricted to cognitively healthy participants, the limited range and variability of the cognitive scores may have impacted our power to detect relationships between the CSF biomarkers and other cognitive tests in our study.

Previous brain imaging studies in healthy adults have found that CSF biomarkers are related to volumetric measures. Healthy elderly individuals from ADNI with low CSF Aβ_42_ levels have significantly higher whole brain loss over time, ventricular expansion, and greater rates of hippocampal atrophy [14]. Fjell et al. [13] also examined CSF biomarker and brain relationships in healthy elderly individuals from ADNI. Lower Aβ_42_ correlated with 1-year change in several regions including putamen, thalamus, superior temporal cortex, cingulate, and frontal brain regions, whereas higher T-Tau/Aβ_42_ was related to atrophy in amygdala, paracentral cortex, and ventricular regions. Higher P-Tau was related to 1-year change in amygdala, paracentral cortex, posterior cingulate, and pallidum. Fjell et al.’s study is also one of the few to show a relationship between P-Tau and cerebral white matter, with higher P-Tau predicting lower white matter volume. P-Tau reported in these studies was assayed for tau phosphorylated at threonine 181, although a similarly aged healthy cohort evaluated at baseline and 2 years later by Glodzik et al. [11] has shown that P-Tau_231_ is also associated with longitudinal medial temporal lobe volume decline and memory function.

In our study of adults with parental family history of AD and a high frequency of APOE4, we found relationships between CSF measures and DTI in several brain regions shown in previous volumetric studies of healthy participants, including temporal, cingulate, and frontal cortices. We did not find a relationship between the CSF biomarkers and gray matter volume (indexed by T1-weighted imaging) in any brain regions, possibly due to the younger age of our cohort in whom tissue changes are likely to be more subtle and less easily detected with volumetric imaging. Using DTI, we had the additional advantage of being able to evaluate potentially subtle effects in white matter, and these relationships were robust and widespread.

### Limitations

While the results of this study are promising, potential limitations should be noted. We adopted a voxel-wise approach in order to assess the extent to which CSF biomarkers were related to brain microstructure and volume across gray and white matter tissue. While we took great care to evaluate for potential errors during registration of the DTI maps to a common template, methods that employ registration to a template may be susceptible to subtle error and statistical effects may be influenced by the size of smoothing filters used in voxel-wise analyses. As a further comment on the DTI results, we would like to note that this study used a tensor model which resolves a single fiber direction within each voxel. We found very robust results when analyzing the MD, axial, and radial diffusivity maps, compared to the FA maps. Due to the complex architecture of white matter, employing FA derived from the tensor model may not have captured the full information available with alternative methods of DTI acquisition and higher order modeling [123]. We should also note that our population was composed solely of participants with parental family history of AD and the frequency of APOE4 was 28%. While this group is at increased risk for AD and thus provides a powerful cohort for examining AD biomarkers, the lack of family history negative participants and high frequency of APOE4 potentially limits the generalizability of our results to a healthy population in addition to limiting our ability to measure relationships between CSF biomarkers and brain measures that are independent of AD risk. Additionally, although our study was longitudinal in the sense that baseline CSF was collected followed by MRI acquisition approximately three and a half years later, simultaneous MRI and CSF collection over two or more time points would have yielded greater power to map out longitudinal change in these measures. Based on the results of this study, additional longitudinal work is warranted. Finally, while our results begin to address the pattern of white matter changes that may occur in the earliest stages of AD, employing white matter biomarker assays in larger cohorts of older adults is expected to clarify the nature of white matter changes in the earliest disease stages.

### Summary

CSF biomarkers previously associated with AD are related to brain tissue microstructure. This study supports an emerging theory that brain white matter alterations are an early event in AD pathogenesis. The mechanisms that underlie white matter alterations and the nature of these alterations deserve further study.

## Supporting Information

Table S1 **Regions where CSF biomarkers were significantly correlated with axial and radial diffusivity in the voxel-wise analyses.**
(DOC)
