# Supplementary material for: CSF T-Tau/Aβ42 Predicts White Matter Microstructure in Healthy Adults at Risk for Alzheimer’s Disease
Source: PLoS One. 2012 Jun 6;7(6):e37720. doi: 10.1371/journal.pone.0037720 (PMC3368882; doi:10.1371/journal.pone.0037720)
Supplement: Table S1 — Regions where CSF biomarkers were significantly correlated with axial and radial diffusivity in the voxel-wise analyses. (DOC) [file pone.0037720.s001.doc]

**Table S1** Regions where CSF biomarkers were significantly correlated with axial and radial diffusivity in the voxel-wise analyses

|  | **MNI Coordinates**  **x y z** | **Peak T Value** | **k (mm^3^)** |
| --- | --- | --- | --- |
| **T-Tau & Axial Diffusivity** |  |  |  |
| R Precuneus WM | 26 −61 42 | 7.74 | 15260 |
| L Middle Frontal Gyrus | −22 45 −12 | 6.54 | 758 |
| R Middle Temporal Gyrus | 60 −40 1 | 5.04 | 285 |
| L Frontal Lobe WM | −18 −4 61 | 4.97 | 128 |
| L Superior Temporal Gyrus WM | −62 −23 7 | 4.41 | 108 |
| L Temporal WM | −54 −13 −23 | 4.37 | 124 |
| R Middle Temporal Gyrus | 46 −63 −4 | 4.26 | 110 |
| R Fusiform, WM | 38 −71 −21 | 4.11 | 85 |
| R Uncus | 20 11 −28 | 3.90 | 90 |
| L Parietal Lobe WM | −32 −37 41 | 3.52 | 55 |
| R Putamen | 24 9 11 | 3.49 | 54 |
| R Medial Frontal Gyrus | 8 −11 68 | 3.40 | 53 |
|  |  |  |  |
| **T-Tau & Radial Diffusivity** |  |  |  |
| R Fusiform | 26 -59 42 | 6.85 | 60480 |
| L Middle Occipital Gyrus WM | -22 -93 16 | 6.57 | 33740 |
| L Precuneus | -36 -69 43 | 6.54 | 369 |
| R Medial Frontal Gyrus | 18 5 54 | 5.64 | 329 |
| R Middle Temporal Gyrus | 58 -42 -1 | 5.44 | 132 |
| L Frontal Lobe WM | -18 -4 59 | 5.11 | 136 |
| R Inferior Parietal Lobule, WM | 48 -41 40 | 5.10 | 222 |
| L Middle Temporal Gyrus WM | -52 -49 -4 | 4.80 | 188 |
| R Putamen | 26 -9 7 | 4.78 | 150 |
| R Posterior Cingulate WM | 16 -48 2 | 4.73 | 298 |
| L Superior Temporal Gyrus WM | -60 -23 7 | 4.65 | 88 |
| L Middle Frontal Gyrus | -24 43 -14 | 4.63 | 86 |
| L Temporal Lobe WM | -52 -13 -21 | 4.26 | 106 |
| R Superior Temporal Gyrus WM | 60 -24 -1 | 4.16 | 79 |
| R Uncus | 20 9 -27 | 4.03 | 104 |
| R Middle Frontal Gyrus | 28 45 -10 | 3.94 | 97 |
|  |  |  |  |
| **P-Tau & Axial Diffusivity** | × | × | × |
|  |  |  |  |
| **P-Tau & Radial Diffusivity** | × | × | × |
|  |  |  |  |
| **Aβ_42_ & Axial Diffusivity** | × | × | × |
|  |  |  |  |
| **Aβ_42_ & Radial Diffusivity** | × | × | × |
|  |  |  |  |

| **T-Tau/Aβ_42_ & Axial Diffusivity** |  |  |  |
| --- | --- | --- | --- |
| R Precuneus WM | 24 -60 38 | 6.87 | 9469 |
| L Superior Frontal Gyrus | -22 43 -22 | 6.86 | 379 |
| R Medial Frontal Gyrus WM | 10 48 -16 | 5.80 | 261 |
| L Uncus | 20 11 -28 | 5.71 | 222 |
| R Middle Temporal Gyrus | 48 -63 -4 | 5.17 | 184 |
| L Superior Temporal Gyrus | -42 13 -22 | 4.92 | 138 |
| R Middle Temporal Gyrus | 58 -42 -1 | 4.86 | 98 |
| L Temporal Lobe WM | -52 -11 -23 | 4.68 | 263 |
| R Superior Temporal Gyrus WM | 58 -24 5 | 4.57 | 144 |
| L Superior Temporal Gyrus WM | -62 -43 14 | 4.55 | 92 |
| L Superior Temporal Gyrus WM | -62 -21 7 | 4.28 | 87 |
| R Middle Temporal Gyrus WM | 56 -14 -16 | 4.24 | 62 |
| L Middle Cingulate Gyrus | -6 22 29 | 4.13 | 285 |
| R Temporal Lobe WM | 42 -6 -31 | 4.12 | 114 |
| L Frontal Lobe WM | -30 -37 39 | 4.09 | 68 |
| R Precentral Gyrus | 32 -20 49 | 4.01 | 81 |
| R Cerebellum, Anterior Lobe | 24 -37 -21 | 3.72 | 79 |
| R Lingual Gyrus | 28 -61 -6 | 3.71 | 52 |
| R Middle Temporal Gyrus WM | 54 -70 23 | 3.69 | 184 |
|  |  |  |  |
| **T-Tau/Aβ_42_ & Radial Diffusivity** |  |  |  |
| L Temporal Lobe WM, ILF | -44 -36 -13 | 7.29 | 98110 |
| R Uncus | 20 9 -27 | 6.16 | 291 |
| R Insula WM | 36 26 11 | 5.67 | 178 |
| L Insula WM | -42 13 -1 | 5.04 | 137 |
| R Middle Temporal Gyru | 58 -42 -1 | 4.93 | 98 |
| R Middle Temporal Gyrus | 46 -39 39 | 4.83 | 153 |
| R Superior Temporal Gyrus | 60 -26 -1 | 4.73 | 132 |
| R Superior Temporal Gyrus | -60 -21 7 | 4.62 | 109 |
| L Putamen | -24 14 0 | 4.54 | 80 |
| R Middle Temporal Gyrus | 48 -63 -4 | 4.53 | 66 |
| L Middle Frontal Gyrus WM | -24 41 -13 | 4.53 | 76 |
| L Superior Temporal Gyrus WM | -62 -43 14 | 4.30 | 85 |
| R Parahippocampal WM | 42 -6 -31 | 4.28 | 184 |
| R Temporal Lobe WM | 32 -76 24 | 4.21 | 284 |
| R Putamen | 26 -9 7 | 4.05 | 100 |
| R Medial Frontal Gyrus WM | 12 46 -16 | 3.97 | 105 |
| R Middle Temporal Gyrus WM | 56 -14 -14 | 3.88 | 61 |
| R Occipital Lobe WM | 28 -59 -4 | 3.87 | 55 |
| R Cerebellum, Anterior Lobe | 24 -37 -21 | 3.82 | 108 |
| R Precentral Gyrus WM | 34 -22 51 | 3.81 | 87 |
|  |  |  |  |
| **P-Tau/Aβ_42_ & Axial Diffusivity** | × | × | × |
|  |  |  |  |
| **P-Tau/Aβ_42_ & Radial Diffusivity** | × | × | × |
|  |  |  |  |
| **NFL & Axial Diffusivity** | – | – | – |
|  |  |  |  |
| **NFL & Radial Diffusivity** | – | – | – |

MNI: Montreal Neurological Institute; k: cluster size; T-Tau: Total Tau; P-Tau: Phosphorylated Tau; WM: White Matter; L: Left; R: Right

× No relationship with any regions at FDR corrected threshold p < .05

– No group differences in any region at FDR corrected threshold p < .05
